# Questions and Challenges Associated with Implementing the Planetary Health Diet: A Narrative Review

**DOI:** 10.3390/foods15213813

**Published:** 2026-10-25

**Authors:** Bożena Regulska-Ilow, Dorota Różańska

**Affiliations:** Department of Dietetics and Bromatology, Wroclaw Medical University, 50-556 Wroclaw, Poland; bozena.regulska-ilow@umw.edu.pl

**Keywords:** planetary diet, environment, nutrient deficiencies, bioavailability, greenhouse gas emission

## Abstract

Global warming has necessitated the development of new nutritional principles that protect both human health and the environment. Both of these conditions are met by a planetary diet, which is dominated by plant-based foods with a small proportion of animal products. A low proportion of animal products carries the risk of nutrient deficiencies, such as calcium and iron, which are highly bioavailable from dairy products and meat, respectively. Consequently, nutritional education is becoming increasingly important, particularly in terms of meal composition, which can enhance the bioavailability of plant-derived nutrients and help prevent deficiencies in essential minerals. Another issue is the implementation of measures to protect populations in low-income countries, where widespread malnutrition could be alleviated by introducing foods fortified with nutrients that are insufficiently supplied by the diet. The risk of malnutrition affects all age groups at high risk of nutritional deficiencies, including children, women, adolescents, and the elderly. The purpose of this narrative review is to present the current body of scientific evidence concerning the association between the EAT–Lancet Planetary Health Diet and health, environment, the risk of insufficient nutrients content in this diet, difficulties with implementation of this diet in low-income countries, and the importance of fortified food product intake, as well as the need for nutritional education in different populations.

## 1. Introduction

Current climate changes are associated with greenhouse gas (GHG) emissions caused by human activity. The global consequences of this phenomenon are independent of the area in which it occurs. Available data show that food systems are responsible not only for about 25% of GHG emissions but are also associated with 70% of freshwater use, 40% of land use and 78% of eutrophication [1]. All of these consequences together caused by food production have a negative influence on the environment and this problem must be reversed [1,2].

Until now, diet has been considered in the context of its relationship with health, without taking into account its impact on the environment. Global warming and its consequences for humans and the environment are forcing a shift in this approach. The transition to a healthy and simultaneously sustainable diet should be supported globally and should include optimizing agricultural practices, resource efficiency, reducing food loss and waste, improving food affordability, and improving the availability of nutritious food with minimal environmental impact [3].

In order to reverse the negative environmental impact of food systems, in 2019 a group of experts from various fields constituting the EAT–Lancet Commission proposed the composition of a Planetary Health Diet (PHD) appropriate for human health and, at the same time, sustainable for the environment. The PHD is based on plant foods, such as grains, vegetables (with only a small part consisting of starchy vegetables), fruits, legumes, and vegetable oils. In this diet, only limited amounts of animal-based foods are allowed (eggs, poultry, dairy products, non-trawling fish, and land animal meat). Sugar is permitted in limited amounts in populations where it is habitually consumed and helps in balancing the dietary energy intake. The team of researchers that developed the diet’s composition specified the intake ranges for each food group, expressed in g/day per 2500 kcal, together with their energy value. The proposed proportions of energy derived from specific food groups allow for a balanced diet while simultaneously minimizing environmental impact in terms of carbon and water footprints and land use. The PHD can be used from the age of 2 years [4]. The new 2025 EAT–Lancet Commission report updates the 2019 recommendations. It advocates a transition from the current food system to one that simultaneously protects human health, operates within planetary boundaries, and ensures equitable access to food and decent living conditions for all. Simultaneous action is needed in three areas: a healthy diet, more sustainable food production, and reduction in food waste. A healthy diet does not have to be more expensive than current dietary patterns; however, social protection, adequate wages, and financial support are essential for people living in poverty. The Commission emphasizes the need for cooperation among governments, farmers, businesses, scientists, civil society, and the financial sector. The report emphasizes the importance of ensuring adequate nutrient intake through the PHD. However, it does not mean that every possible version of the diet automatically meets all individual nutritional requirements. The PHD provides sufficient amounts of most nutrients, although some, such as iron and calcium, warrant particular attention. This applies particularly to women of childbearing age and young children, for whom a higher intake of animal-based products is permitted. The report also indicates that, in vegetarian and vegan diets, the bioavailability of nutrients, as well as fortified foods and supplementation, must be taken into account. Nevertheless, the report does not necessarily ensure that individual nutritional requirements will be fully met by this diet without appropriate dietary planning. However, with regard to nutrient supply, the quantities of foods consumed, the bioavailability of micronutrients, and the nutritional requirements of the study population should be always taken into account. Furthermore, the reference values from the new report are based on an average energy requirement for adults of approximately 2400 kcal/day, and not all food groups have identical reference intake ranges as in the report from 2019. It is also essential to distinguish between two types of evidence: model-based estimates of dietary composition derived from food composition tables and empirical studies assessing actual dietary intake and biomarkers. The results of these methods do not have to be equal [5].

Many authors from different countries have assessed the adherence of typical diets and eating patterns with the Planetary Health Diet. The publications cited in this review refer to indices developed based on the original version of the Planetary Health Diet (2019), as the revised recommendations were not published until late 2025. Only Gimpfl et al. [6] compared two versions of the index: the PHDI-2019, based on the original EAT–Lancet model published in 2019, and the PHDI-2025, which incorporates the updated EAT–Lancet recommendations published in 2025. The updated version introduced several modifications, including changes to the recommended intake ranges for certain animal-source foods and the addition of sodium as a health-related component. The authors demonstrated near-perfect agreement between the two indices, suggesting that the update represents a refinement rather than a fundamental redefinition of the PHD model.

The association between the PHD and the risk of diabetes [7], obesity in different age groups [8,9] cardiovascular diseases [7,10,11,12], hypertension [7,13,14], cancer [15,16], and mortality [17,18] has been assessed.

Some authors also paid attention to how to adapt the diet for people with various diseases (chronic obstructive pulmonary disease [19], liver diseases [20], chronic kidney disease [21]) in order to contribute as little as possible to environmental devastation. The Planetary Health Diet was also assessed taking into account its association with carbon footprint [6,22], water footprint [23,24], and land use [23,24]. However, only a few authors have paid attention to the risk of insufficient nutrient intake when adapting the current diet to the PHD [25,26,27].

The PHD is universal because it does not have to be the same across the globe. The proposed ranges of individual food groups allow for the adjustment of the diet’s composition to dietary habits, product availability, climatic conditions, and people’s incomes [28,29].

The EAT–Lancet Commission assumed some flexibility in adapting to the PHD rules, caused by the fact that in some populations, their dependence on agriculture and animal protein for subsistence needs to be taken into account. In populations with a high prevalence of malnutrition, it is difficult to obtain adequate amounts of micronutrients derived mainly from plant-based foods [30]. Therefore, especially in the first period of transformation, it is necessary to carefully consider in every aspect the real sources of plant- and animal-based foods in the diet that function as the suppliers of macro- and micronutrients.

The PHD is a challenge for global institutions and governments. Transforming traditional diets into a planetary healthy diet in different regions of the world, on different continents, will require significant financial investment to implement dietary changes. The primary goal of its implementation is to restrict global warming to below 2 °C and to feed the dynamically growing population, which may reach 10 billion people by 2050. Broadly speaking, the global transformation means that the consumption of healthy foods such as fruits, vegetables, legumes and nuts must be doubled, and simultaneously a greater than 50% reduction in the global consumption of unhealthy foods such as added sugars and red meat is needed. The expected reductions or increases in the consumption of specific food groups are unevenly distributed across different regions of the world. In the rich countries of North America and Europe, the pressure will be put on the significant reduction in the consumption of animal-based foods (red meat, poultry, eggs, dairy products) and starchy vegetables, but also on the higher consumption of plant-based foods (vegetables, fruits, nuts, whole grains, legumes) and fish. In contrast, in Sub-Saharan Africa, only the consumption of starchy vegetables must be reduced by several times, while red meat consumption is at an appropriate level. However, the intake of all other food groups must increase. The PHD appears to be useful for high-income countries but problematic for low-income countries [4,25,30,31].

The aim of this narrative review was to present the current body of scientific evidence concerning not only the association between the EAT–Lancet Planetary Health Diet and health and environment but also the risk of insufficient nutrient content in this diet, difficulties with implementation of this diet in low-income countries, and the need for fortified food product intake, as well as the need for nutritional education in different populations. What distinguishes this review from other studies is its comprehensive discussion of issues related to the concept of the PHD, which may serve as inspiration for further efforts aimed at introducing this diet among various populations, taking into account the specific characteristics of local food availability as well as the economic challenges faced by different countries, and to highlight the importance of fortified foods in improving dietary nutrient adequacy.

## 2. Materials and Methods

A search of the existing literature was undertaken in July 2026 and was based on the PubMed electronic database. The following search terms were used to identify the relevant literature—“EAT-Lancet” or “Planetary Health Diet”, appearing in the title/abstract together with one of the following words: “sustainability”, “carbon footprint”, “water footprint”, “shortfalls”, “mortality”, “cancer”, “cardiovascular disease”, “diabetes”, “hypertension”, “obesity”, “risk factor”, “risk factors”. The total number of articles found was 508. Duplicate papers were excluded; next, abstracts and full texts of the remaining articles were reviewed to assess their relevance to the scope of this study. Finally, 59 articles were included in this review (Figure S1). In addition to the publications identified through the search strategy described above, 18 additional publications were included to address specific aspects of the topic that were not sufficiently covered by the search results, such as nutrient bioavailability. These publications were identified based on their thematic relevance and the use of appropriate keywords, without applying any temporal restrictions to the search.

## 3. Adapting the Diet of Children and Adolescents to the Assumptions of the Planetary Health Diet

To ensure that the shift to the PHD will take place naturally, without drastic dietary changes, it was proposed that the development of taste preferences should begin in infancy [31] and in older children [32], taking into account their nutritional needs. Complementary solid foods in the second half of the first year of life could be the basis for future nutritional choices in terms of taste. Two periods for implementing a balanced diet in early childhood are proposed: at 6 and 24 months of age [31].

In a German study about older children, adapting their usual diet to the PHD resulted in an increase in the Planetary Health Diet Index (PHDI) from 68.24 to 81.51 points (maximum score was 150 points). Taking into account maintaining the nutritional needs of children, this increase was achieved by increasing the intake of fruit, vegetables, fish and seafood, vegetable oils, and chicken (or substitutes) and, in contrast, by reducing the intake of tubers and potatoes, dairy products, red meat, animal fat and added sugar. The main factors contributing to increasing the adherence to the PHD, easy to implement in practice, were identified, such as increased consumption of peanuts, vegetables, and whole grains and reduced consumption of tubers and potatoes and red meat. It was shown that the reference diet for children and adolescents can be adapted to the PHD model in institutional catering facilities. Most of all, the researchers demonstrated that reducing the intake of meat in favor of legumes in cooked meals allows for meeting nutrient requirements. The changes that made this possible included adding nuts to muesli instead of cornflakes, replacing dark green bitter vegetables with other sweeter raw vegetables more acceptable to younger children, and replacing potatoes with pasta in salads. Balancing children’s diets requires a threefold increase in the consumption of nuts and seeds, either unprocessed or processed (peanut butter or hummus). The authors suggested that the environmental sustainability of the diet could be further improved by replacing cow’s milk with plant-based alternatives fortified with key nutrients, including calcium and vitamins B12 and B2. They also discussed the possibility of allowing added sugars to contribute up to 10% of total energy intake to maintain the diet’s palatability for children, while acknowledging the importance of limiting added sugar consumption. Adequate water intake, especially as tap water, was emphasized as a priority component for maintaining physiological water balance, especially in children, although it was not included in the PHD recommendations. The carbon footprint of tap water is lower than that of packaged water. The authors found that the transition from the reference diet to the PHD was associated with relatively small changes in food costs. Adapting the German dietary guidelines for children to the PHD could also lead to similar considerations in other countries [32].

On the other hand, some problems in adapting the current diet of Finnish preschoolers to the reference PHD were observed because it would require drastic changes in children’s eating habits and also in Finland’s food system. Children would need to eat more legumes, nuts, vegetables and whole grains and less red meat, dairy products and sugar [33]. Another study showed that the diet of preschool children from Poland contained insufficient amounts of calcium, iron and vitamin D, but on the contrary, contained excess amounts of protein, vitamin A, salt and sugar. None of the five facilities assessed in Poland fully implemented the principles of the PHD. It is important to note that the menus did not limit meat consumption, did not include seasonal and regional vegetables and fruits, nor provided sufficient amounts of whole-grain products and legume-based dishes [34].

The diet of Mexican teenagers was far from the composition of the PHD, but it was computed that reducing the intake of animal protein (which was consumed doubly) and increasing the consumption of whole grains would probably have a positive association with environmental sustainability, as well as academic performance [35]. Other authors observed that the PHD among teenagers was associated with better anthropometric parameters and quality of life [36]. Lu et al. [37], based on the observational study, assessed the PHDI-green pattern among adolescents in China. They were considered adherent to the PHDI-green pattern if they consumed fresh fruits and vegetables daily, including at least one type of each. The results of this study suggest that greater adherence to the PHDI-green pattern among the study group may be associated with lower risk of obesity and better mental health [37].

After analyzing dietary data from 19,601 adolescents in six Latin American countries, adherence to the PHD was found to be low (42.4 points, 95% CI 42.0–42.8), with lower adherence in urban compared to rural areas. Higher adherence to the PHD was observed among adolescents from lower socio-economic backgrounds than those from higher socio-economic backgrounds. Higher adherence was associated with lower ultra-processed food intake [38]. The results of the studies on the Planetary Health Diet among children and adolescents are presented in Table 1.

## 4. Adapting the Diet of Adults to the Assumptions of the Planetary Health Diet

Appropriate micronutrient intake is a key point in a healthy balanced diet. Therefore, Italian researchers showed strategies aimed at adapting the Mediterranean diet to the PHD, taking into account the adaptation of this pattern to different energy values. A modified diet (the PHD adapted to the Mediterranean diet) with an energy value of 2500 kcal/day meets all nutritional requirements, except for vitamin D. It was also acceptable taking into account the impact on the environment because its carbon footprint was higher only by 12%; however, its water footprint was lower by 6.3%. For the diets with lower energy values (1600–2500 kcal/day), the aim was reached based on modifying the intake of different food groups to ensure the appropriate nutritional value. The proportion of some animal-based foods was increased (e.g., dairy products, seafood, eggs), but the intake of others (not only animal-based) was decreased (e.g., red meat, legumes, nuts, added fats). These changes allowed them to maintain an appropriate intake of heme iron and calcium. About 1000 mg of calcium was derived by the 2L of tap water (19%), dairy products (35%) and the other sources. About 18 mg of iron was derived by grains (34%) and vegetables (22%). The reference intake of dietary fiber was assumed as 16.7 g/1000 kcal, and this amount was derived by grains, fruits, vegetables, legumes and nuts (38%, 21%, 20%, 10% and 7%, respectively). The modifications made to adapt the Mediterranean diet to the PHD were found to be difficult when taking into account the appropriate nutritional value of the diet. The biggest challenge was observed in case of iron and zinc due to the reduction in grain product intake, which was necessary to decrease the intake of energy, dietary fiber and phytates. The content of heme iron was almost at the same level because the animal-based foods were only slightly reduced. The absolute amounts of saturated fat and protein decreased but were still in accordance with the recommendations. The environmental impact of the diet adopted on 2000 kcal was lower by 4.7% (−0.13 kg CO_2_ eq/day) and 11.5% (−374 L/day) for carbon and water footprint, respectively, in comparison to a diet with 2500 kcal. The highest differences were observed between the diets adopted for the 1600 kcal and 2500 kcal diets (−0.4 kg CO_2_ eq/day and −831 L/day for carbon and water footprint, respectively). It was also emphasized that sustainable diet with energy intake below 2500 kcal/day can be useful for people with overweight, obesity and non-communicable diseases [3].

However, it is important to emphasize that there are different problems and challenges in low-income countries (low Gross Domestic Product per capita) associated with implementation the PHD, which was analyzed by de Pee et al. [30] in a study conducted in Indonesia. In low-income countries, the crucial problem is the prevalence of malnutrition related to the insufficient intake of nutrients, food prices, and the lack of products fortified with these nutrients. The possibility to compare habitual diets with the PHD is also limited by the lack of data on food consumption and food supply. de Pee et al. [30] used the FAO food balance sheet, Indonesia Household Income and Expenditure Survey household food expenditure, food composition, life cycle assessment, food losses, and trade data in order to compare habitual diets with more optimized plant-based diets and the PHD. The habitual diets were characterized by limited dietary diversity and inadequate intake of several essential nutrients, and therefore it is not obvious if the sustainable nutrition, health, affordability and environmental protection goals can be simultaneously achieved in low-income countries. To reach nutritional adequacy, animal-based foods were necessary. The costs of all diets exceeded the national average food expenditure, with the exception of optimized diets. However, diets optimized for nutrients and cost best met nutritional needs but were associated with high greenhouse gas emissions. Moreover, none of the thirteen dietary scenarios that were analyzed achieved optimal outcomes regarding nutrition, health, affordability, and climate goals simultaneously. Undernutrition in early years of life affects almost 31% of children in Indonesia; therefore, improving diet quality is essential for preventing the adverse consequences of malnutrition in childhood and reducing their long-term effects in adulthood. The diets in Indonesia are rich in rice and products with high content of sugar, unhealthy fats and salt. To meet the goals both for health and the environment in low-income countries, the consumption of rice and unhealthy food should be decreased. Food fortification may help to achieve higher nutritional value of the diets. For example, rice, wheat flour or cooking oils can be fortified with nutrients that are commonly underconsumed [30].

Australian researchers found that a healthy and sustainable diet was more affordable than the typical Australian diet for people living in a metropolitan area. It was assessed that for the PHD they should spend about 13% of their weekly income instead of 16% for a typical diet. What is interesting is the authors used kangaroo meat in calculations because of its relatively low impact on the environment [39].

A study conducted in a group of young American adults showed that their diet was not consistent with the PHD (only 4.1 ± 1.4 points, on a scale of 0–14 points). Two factors which were mainly associated with the PHD were home availability of healthy food and lower frequency of fast-food consumption. Moreover, participants with higher socio-economic status and higher level of education achieved better results [40].

Cámara et al. [41] analyzed the cost and affordability of the current diet in Argentina, comparing it with a diet based on national dietary guidelines and three diet variants based on the EAT–Lancet recommendations. It was computed that diets based on national dietary guidelines were, on average, the most expensive (least affordable), while the current isocaloric diet and a flexitarian diet with fewer animal protein sources were the cheapest (most affordable). This suggests that there are dietary patterns that can reduce greenhouse gas emissions and provide health benefits without increasing food expenditures [41].

The feasibility and costs of implementing the PHD in Chile compared to the current diet were analyzed. Its adaptation to the lowest income quintile in Chile was taken into account, which would also allow for its implementation by the poorest population group. The difference in the costs of a healthy and sustainable basic food basket and a basic food basket varied seasonally, ranging from 4.6% to 21.1%. The cost of a healthy and sustainable diet, that provided 2001 kcal/day, was on average 13.9% higher than the basic food basket, showing a significant economic barrier for low-income individuals [42].

In a group of older adults in Iran, socio-economic status (SES) and food prices were also found to be significantly associated with food choice and diet quality. Adherence to the PHD was associated with demographic group. Higher adherence was associated with lower daily dietary cost [43].

Adherence to the PHD was assessed as poor in 37 African countries, in terms of carbon footprint, water footprint, and land use. However, given that few African countries have dietary guidelines, the PHD can provide a basis for improving health and environmental sustainability through smart food policies [44].

Swedish researchers estimated, taking into account Swedish culinary culture, that dietary changes that would reduce environmental impact by 30% compared to current diets are feasible. Analyzing various scenarios (reflecting technological development prospects, behavioral changes, import trends, and values), none of the scenarios achieved climate targets without eliminating energy-related emissions in the food chain. However, for dietary changes to impact environmental and health goals, improvements in production and waste reduction are necessary [45].

The results of the studies on the adherence to the Planetary Health Diet among adults are presented in Table 2. The results of individual studies may vary not only due to differences in the study groups (e.g., age, energy requirements or socio-economic status) but also due to geographic, economic, and climatic conditions of each country. Geographic location may contribute to this variability, as the nutritional value of foods available in different countries can differ due to factors such as soil mineral composition. Differences in the level of economic development and local economic conditions may further affect food affordability and availability. In addition, depending on the climate of a given country, crop cultivation and livestock production may have different environmental impacts, for example, due to the need for irrigation in areas with limited rainfall or for adequate cooling and storage conditions in regions with high temperatures.

## 5. Risk of Inadequate Nutrient Intake During Adapting Traditional Diets to the PHD in Different Populations

Studies conducted to compare typical habitual diets with the PHD in different populations on different continents showed inadequate intake of a number of nutrients or the risk of inadequate intake following a drastic reduction in the consumption of animal products. For example, adapting the Australian diet to the PHD would require, among others, decreasing the intake of dairy products while 20-fold increasing the intake of legumes. This would result in decreasing the percentage of EAR (Estimated Average Requirement) for calcium to 71%, compared to the 101% observed in the average Australian diet [46]. Adherence to the PHD among adults in Turkey was low, and lower adherence to the PHD was observed among participants with higher BMI. The average PHD index was 41.5 points (on a scale of 0–150 points). Higher PHD index indicated higher adherence to the PHD and in this population it was positively associated with the intake of fiber, vitamin E, potassium, and folate, whereas there was a negative association with the intake of pyridoxine and calcium [47]. A study conducted among young Turkish adults also showed that the compatibility between their diets and the PHD was low, despite their high level of education. The results of this study indicate that there is a need to revise both the level and the content of education programs, taking into account the challenges of today’s world [48]. A low adherence not only to the PHD but also to the Finnish recommendations was observed in a study conducted among Finnish adults. There was no association between diet quality and anthropometric changes during a 7-year follow-up [49].

Striving to adapt the composition of the diet to the recommendations of the PHD poses a risk of insufficient nutrient intake especially in high-income countries (for example, in the USA [50] and Europe [51]). Frank et al. [50], analyzing data from NHANES adult participants, found that greater adherence to the PHDI was associated with a lower prevalence of inadequate iron, fiber, and potassium intake. For iron, this prevalence decreased from 4.3% in the lowest PHDI quintile to 3.1% in the highest, whereas inadequate calcium intake increased from 37.1% to 44.3%, highlighting the need to monitor calcium intake in PHD-compliant diets. However, inadequate nutrient intake does not necessarily indicate clinical deficiency, and these findings reflect overall dietary patterns rather than the nutritional value of individual milk alternatives. The latter depends on product composition and fortification, particularly with calcium and vitamin D, and should therefore be assessed on a product-specific basis [50]. The recommendation to reduce the intake of animal products, mainly meat and dairy, poses a risk of iron and calcium deficiencies, respectively [52]. It was observed that individuals with a higher PHD index had a lower risk of insufficient intake of dietary fiber and potassium because of higher consumption of plant-based foods. On the other hand, there is a higher probability of inadequate calcium intake in individuals with higher PHD index [50]. However, the appropriate selection of plant-based foods would help to improve calcium intake because of its high content and bioavailability in some vegetables, seeds or legumes [52].

The bioavailability of calcium from plants varies from high (40–50%) in cruciferous vegetables (kale, broccoli) to lower (15–20%) in legumes [53] and is particularly high in fortified products (plant-based beverages, tofu), often comparable to milk [54].

Data from the Global Burden of Disease (GBD) 2019 showed that micronutrient deficiency is prevalent worldwide and about one-third of people have at least one form of micronutrient deficiency. The most prevalent are iron, vitamin A, iodine, vitamin B9 and zinc deficiencies, and also vitamin D deficiencies. However, analysis showed that globally the age-standardized prevalence rates of iodine deficiency (assessed based on visible goiter—grade 2 and associated complications, while subclinical deficiency and grade 1 goiter were excluded), vitamin A deficiency (assessed based on serum retinol level below 0.7 µmol/L, blindness and vision loss associated with corneal ulceration and scarring), and dietary iron deficiency (inadequate dietary iron intake, excluding other causes) has decreased since 1990. Worth noting is the prevalence of iodine deficiencies which decreased in some developed countries because of salt iodization. This is a good example of cost-effective possibilities to reduce the burden of micronutrient deficiency at the population level, particularly in countries with low sociodemographic indexes and low healthcare accessibility and quality (HAQ) [55].

The composition of habitual diets in different populations was compared with the Planetary Health Diet recommendations by many researchers. For example, the study conducted in the Swedish cohort showed that the EAT–Lancet diet can supply sufficient micronutrients. Regarding nutrient biomarkers, greater adherence to the EAT–Lancet diet was associated with a lower risk of folate deficiency, although a slight increase in anemia risk was observed among women. No significant associations were found between adherence and the risk of selenium, zinc, or vitamin D deficiency in women. In men, however, higher adherence was linked to a slightly lower risk of vitamin D deficiency. Additionally, one of the dietary scores (seven different EAT–Lancet diet scores were used to measure adherence) was associated with a reduced risk of zinc deficiency. It is worth noting that the authors concluded that the results can be different for various scoring methods and energy-adjustment modeling [56]. However, based on the American analysis, it was pointed out that there might be a risk of insufficient intake of some nutrients associated with decreased intake of sugar, red meat and meat products by more than half. On the other hand, it needs to be clarified if it is possible to compensate for the insufficient nutrient intake caused by lower intake of animal-based foods through the doubled increase in global consumption of fruits, vegetables, nuts and legumes [2].

One of the analyses based on the National Health and Nutrition Examination Survey (NHANES) data from 2003 to 2018 was conducted to assess the relationship between the PHD index and inadequate nutrient intake (among others iron, calcium, potassium, dietary fiber) in a group consisting 33,859 American adults. It was observed that the average PHDI increased from 62.7/140 points in 2003–2004 to 66.9/140 points in 2017–2018. Over the study period, the intake of non-starchy vegetables significantly decreased, while the intake of whole grains, nuts, seeds and unsaturated oils increased. Considering foods that should be consumed in moderate or limited amounts, the intake of poultry and eggs increased over the study period, while the intake of red and processed meat, added sugars, saturated fats and starchy vegetables decreased. It was also observed that higher PHDI values were associated with lower likelihood of having inadequate intake of iron, dietary fiber and potassium; therefore, the shift to the PHD is desirable [50].

## 6. Alternative Solutions in the Plant-Based Food Industry Contribute to a Healthy and Balanced Diet

The Planetary Health Diet is abundant in plant-based foods, but the limited intake of animal-based foods may contribute to insufficient intake of some nutrients which can be found mainly in these types of food. One of the examples is the risk of insufficient calcium intake because of limited intake of dairy products. A possible solution is to choose plant-based beverages (almond, oat, soy, etc.) as an alternative instead of cow milk [57]. However, Biscotti et al. [58] computed that replacing cow milk with different plant-based beverages lead to reduced protein intake (except for not-calcium-fortified soy drinks), as well as to reduced intake of saturated fat, vitamins B2 and B12, and increased intake of fiber. Moreover, replacing cow milk with not-calcium-fortified plant-based beverages decreased calcium intake. This analysis showed that cow milk is a very important source of calcium and therefore, when reducing milk consumption in the PHD in favor of plant-based beverages, attention should be paid to choosing fortified options.

The production of plant-based beverages is a more environmentally friendly process because it results in lower greenhouse gas emissions, as well as lower land and water use. But it needs to be emphasized that these types of products must be fortified with nutrients such as calcium, vitamin B2, B12 or some others to be useful in effectively counteracting insufficient nutrient intake in different populations. Replacing dairy with one serving of a fortified plant-based alternative product would not contribute to significant changes in nutrient content in the diet but would decrease the negative impact on the environment. However, plant-based alternative products are often categorized as ultra-processed foods because they contain stabilizers and emulsifiers, which suggests that they have unfavorable nutritional and health properties. Despite that, taking into account nutritional value (for example, vitamin and mineral content) and lower environmental impact of these foods this classification should be reconsidered [57]. Fortified plant-based alternative foods may help to improve the nutritional adequacy of the PHD by providing nutrients that may be limited when the intake of dairy or meat is reduced. However, their nutritional value varies considerably depending on product composition and degree of processing. Moreover, the relatively high cost of some fortified plant-based products may limit their accessibility and widespread adoption, particularly among lower-income populations.

## 7. Association Between Diet Composition and Gross Domestic Product (GDP)

Chen and Chai [1], based on a threshold regression analysis and data from 147 countries collected in 2018, assessed how the Gross Domestic Product (GDP) per capita influences the differences between current diets and the PHD at the national level. It was found that there is an association between economic conditions and food consumption patterns for all types of food. This analysis allowed us to identify countries that require greater financial support based on the GDP per capita, which can be helpful for policymakers to make more efforts to implement appropriate interventions to improve diet.

It was observed that 11 food groups from the PHD can be divided into three types of food. Vegetables, fruits, dairy products, and nuts were classified as foods that are encouraged to consume, while red meat as foods that are discouraged. Other foods (tubers or starchy vegetables, grains, legumes, poultry, eggs, seafood) are those that should be eaten in appropriate amounts. It was shown that the intake of dairy products (source of calcium and protein) increases with an increasing GDP per capita. However, nut consumption remains insufficient in almost all countries worldwide. Moreover, it is not associated with an increasing GDP per capita. On the other hand, red meat consumption is too high in almost all countries, and it increases with an increasing GDP per capita. Because of the negative impact on health, people should be encouraged to limit red meat consumption. The intake of tubers or starchy vegetables and legumes is inversely associated with the increase in the GDP per capita, in contrary to other food groups, where the increasing consumption is observed with increasing GDP per capita. The analysis conducted by Chen and Cha [1] revealed that the GDP per capita, which amounted to $20,000, is not only a border that distinguishes developing and developed countries but is also almost a threshold for all of the food groups mentioned above.

It was found that diet composition depends on the GDP. The results of this analysis, which was based on data from 147 countries, showed that there are differences between the PHD recommendations and the intake of all food groups, but the changing rates of food intake decrease with increasing GDP per capita [1].

It is also important to report the results of a study conducted by Hirvonen et al. [59]. The authors conducted an analysis of the costs and economic affordability of the EAT–Lancet diet in 159 countries and found that the EAT–Lancet reference diet was unaffordable for a substantial proportion of the global population, particularly in low-income countries. The diet cost more than the total per capita household income of at least 1.58 billion people and was, on average, 60% more expensive than the least-cost diet meeting essential nutrient requirements. Fruits and vegetables accounted for the largest share of total dietary costs. On the other hand, Headey et al. [60] demonstrated that estimates of the cost and affordability of the EAT–Lancet reference diet are highly sensitive to methodological assumptions. Accounting for demographic differences and improving the estimation of household food budgets reduced the estimated number of people unable to afford the diet from 3.02 to 2.13 billion. These findings highlight the importance of methodological choices when comparing dietary cost and affordability estimates across studies.

## 8. The Impact of the Planetary Diet on the Environment

Recently, the authors of numerous studies using the PHDI to assess diet quality in various populations worldwide have also highlighted the relationship between diet and the environment. Most studies have reported associations between adherence to the planetary diet and lower morbidity and mortality, whereas its relationship with the environment is uncertain. For example, in a study of Chinese adults in Singapore, greater adherence to the PHD (a higher score) was associated with a reduced risk of mortality from chronic diseases. Higher PHDI was associated with lower greenhouse gas emissions but a larger total water footprint and land use. Lower greenhouse gas emissions were mainly caused by lower consumption of total grains (primarily refined grains) and red meat. The main grain product for Asian populations is rice. Rice, as a major global crop, emits more greenhouse gases than other cereal crops. Furthermore, adopting a diet free from animal products (especially red meat) could reduce greenhouse gas emissions by an additional 49%. In this study, higher consumption of fruits, dairy products, vegetables, and legumes was associated with a higher PHDI but also with an increased water footprint and greater land use. In contrast, reduced consumption of red meat and total cereals was associated with a higher PHDI, as well as a lower water footprint and reduced land use. Therefore, it seems reasonable to recommend a reduction in the consumption of total cereals and red meat to offset the impact on the water footprint and land use resulting from increased consumption of the recommended dietary components [24].

Taking Thailand as an example where unfavorable nutritional and environmental trends have been observed since the early 1960s, achieving the desired changes towards a healthy diet and environment will require a significant effort. The intake of many food groups exceeded the upper PHD reference limits: sugar (+452%), red meat (+220%), grains (+143%), saturated oils (+20%), and eggs (+19%). On the contrary, vegetables (−63%) and unsaturated oils (−61%) fell below the lower PHD reference limits. This increase in the production and consumption of different food groups has contributed to the environmental devastation, resulting primarily from increased greenhouse gas emissions and the increased use of nitrogen fertilizers, pesticides, and phosphorus, which has had a direct impact on soil degradation, eutrophication, climate change, and biodiversity loss [28].

In a cross-sectional study conducted by Conrad et al. [61], eight dietary quality indicators and seven sustainability indicators were assessed for their sustainability impact on health and the environment. All indicators that emphasized plant-based foods over nutrients or animal-based foods were associated with more favorable sustainability outcomes [61]. Suter et al. [62] also observed that in Swiss diets, lower intake of animal-source products was linked to lower greenhouse gas emissions and land use but, on the other hand, high intake of fruits and vegetables was linked to higher costs and greater risks of negative social impacts.

## 9. Increasing the Bioavailability of Micronutrients Is a Way to Increase the Adequacy of the PHD

It is important to consider various aspects of a plant-based diet. The authors highlight the potential for quantitatively adjusting the intake of plant-based foods based on the bioavailability of nutrients and the impact of other compound on this bioavailability [29].

In this context, three issues are important: the impact of dietary composition on the bioavailability of nutrients, reducing the intake of antinutritional factors, and, above all, education on how to compose a diet with an adequate content of strategic nutrients. A plant-based diet with minimal animal-based products may expose women of reproductive age and young children to iron, zinc, and calcium deficiencies, primarily due to the low bioavailability of these minerals from plant foods and the high content of antinutritional compound such as phytates, oxalic acid, and polyphenols in plant foods. Mineral absorption is hindered by oxalates and phytates. Disseminating knowledge about proven methods for increasing the bioavailability of minerals from plant-based foods, such as grinding, soaking, pressure cooking, sprouting, and fermentation, could be helpful. These technological processes are effective in increasing the bioavailability of minerals from legumes, cereals, and oilseeds and reducing antinutritional factors. They are also routinely used in many populations [29,63].

In the case of calcium, the main factor limiting its bioavailability are oxalates. Therefore, including green leafy vegetables in the diet, such as turnip greens, kale, and bokcho, which have high calcium bioavailability, could significantly improve calcium balance [64]. Calcium intake can also be increased by consuming high-calcium mineral waters, legumes, and calcium-fortified plant-based alternative products [65]. High sodium and protein intake can exacerbate calcium deficiency by increasing urinary excretion [66].

When it comes to iron bioavailability, a distinction should be made between heme iron from animal sources, which has high bioavailability (about 15–35%), and non-heme iron, found primarily in plants but also in milk and eggs, which has low bioavailability at 2–20% and is strongly associated with a meal composition and iron status in the body. In a plant-based diet, the bioavailability of non-heme iron can be increased several-fold by selecting appropriate products. The most potent enhancer of non-heme iron absorption is vitamin C. Therefore, appropriate intake of fruit and vegetable abundant in vitamin C together with tubers and cereals may be a practical approach to reducing the risk of iron deficiency anemia [67,68].

Antinutrients in food, in addition to limiting the bioavailability of minerals, also exhibit beneficial effects. For example, phytic acid and phenolic compounds have antioxidant, anti-inflammatory, and anticancer properties. Their food sources, cereals and legumes, contain protein, iron, and zinc and constitute the main source of dietary energy in many populations.

Klapp et al. [29] also emphasize the importance of local edible plant resources, not only cultivated but also wild, in the context of their environmental benefits and meeting nutritional needs. Brazil and Canada are countries that have integrated indigenous foods into national food-based dietary guidelines. Similar efforts in other countries and on other continents appear to be justified to ensure nutritional security. Furthermore, the authors believe that to eliminate various nutrient deficiencies, different options such as fortification, supplementation, and additives should not be dismissed, as they can play a key role in improving health outcomes. Instead, they should be assessed in terms of costs and benefits to humans, while recognizing the limits of a planetary diet.

## 10. Actions to Implement the Planetary Diet—Educating Young People

Rapid climate change is forcing the search for ways to implement the PHD. One of the examples was a training program for students from various fields of study that was conducted at the University of Göttingen in Germany, combining theoretical knowledge of the Planetary Health Diet’s composition with practical implementation in the kitchen. This pilot study demonstrated improvements in young people’s knowledge and practical skills in implementing the PHD and also demonstrated the potential of students as agents of change for sustainable food systems [69].

American researchers found that food purchases in dining halls at selected American universities are currently not aligned with the PHD. However, the results indicated that universities could potentially benefit from reduced food-related greenhouse gas emissions, improved nutritional metrics, and lower food costs by increasing purchases of plant-based foods aligned with the PHD and reducing meat purchases. This would contribute to improved student eating behavior [70].

The results observed in a study conducted in Croatia also emphasized that there is a need for systemic changes at the university level to support sustainable eating behaviors [71].

## 11. Implementing the Planetary Health Diet in Healthcare and Institutional Settings

It has been shown that there is significant potential to reduce inappropriate meals served to nursing home residents and simultaneously promote the adoption of a Planetary Health Diet. Adopting the guidelines aligned with the PHD can lead to more sustainable food production and consumption and also provide health benefits. Acceptance of these guidelines would create a possibility to establish a new standard of care while also having a positive impact on the environment [72].

Serving meals that adhere to the PHD guidelines in institutional catering facilities such as restaurants, cafeterias, and long-term care homes [73] can help accelerate the shift to a healthier and more balanced diet through a system for ordering specific products for preparing meals [74,75].

Among the many proposed actions aimed at reducing land use and limiting the negative impact on biodiversity in the food system, reducing food waste seems most important. Other authors emphasize that reducing the consumption of one food group requires increasing the consumption of another, which can shift biodiversity threats to another region. Therefore, the priority should be the widespread implementation of food waste-reduction initiatives, which may generate less resistance and have fewer unintended consequences than other actions [76].

Night-shift healthcare workers represent a group of professionals who, despite their knowledge of health factors, make poor dietary choices due to a lack of access to healthy food in the work environment. It seems that relatively simple actions for increasing the accessibility of healthy food in the work environment would be crucial for better diet quality and environmental sustainability [77].

## 12. Summary and Limitations

**Figure 1:**
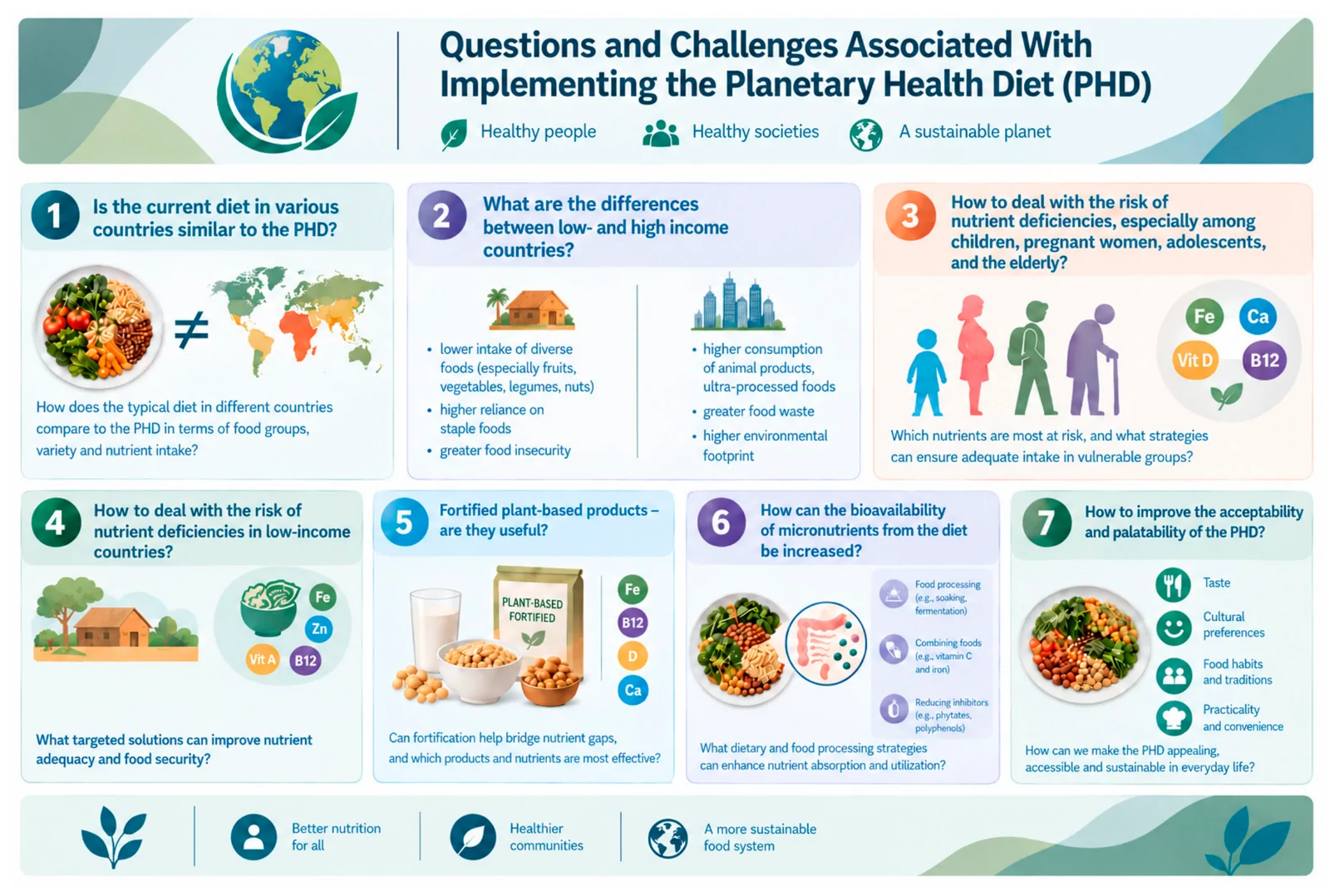
Questions and challenges associated with implementing the Planetary Health Diet. The figure was generated by ChatGPT (OpenAI) version GPT-5.6 Luna based on the authors’ specifications.

The review herein provides an overview of the current scientific literature concerning not only the association between PHD and health and environment but also the risk of inadequate nutrient content in this diet, difficulties with implementation of this diet in low-income countries, and the need for fortified food product intake, as well as the need for nutritional education in different populations. The Planetary Health Diet was proposed in 2019 and interest in this topic has grown substantially in recent years, as reflected by the increasing number of publications. Since 2019, numerous studies have confirmed the association between PHD and health outcomes. However, numerous gaps remain to be addressed in future studies (Figure 1).

Based on the evidence reviewed in this manuscript, adapting children’s and adolescents’ diets to the Planetary Health Diet (PHD) should take into account age-specific nutritional requirements, taste preferences, and the development of healthy dietary habits from early childhood. The studies discussed above suggest that dietary adherence to the PHD can be improved while maintaining selected indicators of nutritional adequacy. However, the available evidence does not necessarily establish that all nutritional requirements are met across different stages of growth and development. Implementing the PHD may also present challenges related to established dietary habits and the potential for inadequate intake of certain nutrients. Therefore, dietary modifications should be age-appropriate and gradual, with particular attention to energy intake, nutrient adequacy, and children’s and adolescents’ acceptance of the foods included in this dietary pattern.

Another important consideration is the role of economic disparities, particularly differences in economic development and income levels across countries. It was observed that adapting the PHD to different energy levels and economic conditions is possible, but it requires changes in the proportions of specific food groups. The PHD can simultaneously support health and reduce the environmental impact of nutrition; however, its implementation depends on energy requirements, food availability, economic situation, and local dietary habits. These economic and contextual factors are also essential when interpreting differences in the reported costs of the PHD across countries and populations.

Differences in the reported costs of the Planetary Health Diet may reflect variations in food prices, study populations, and methodological approaches. While some studies estimate the minimum cost of meeting predefined dietary targets, others assess actual dietary intake or compare the PHD with alternative dietary patterns. These approaches may yield different results, as theoretical minimum-cost diets do not necessarily reflect real purchasing behavior, food availability, or consumer preferences. Moreover, dietary cost differs from affordability, which depends on household income and essential expenditures. Differences in energy requirements and demographic characteristics may also contribute to variation in estimates. Thus, conflicting findings may reflect differences in populations, reference diets, and analytical methods rather than the inherent cost of the PHD [59,60].

A further aspect is the content of micronutrients in the PHD. The studies presented in this review comparing the habitual diets of various populations with the PHD indicate that its adoption can yield environmental benefits and improve the intake of certain nutrients, particularly fiber, potassium, folate, and vitamin E. At the same time, a significant reduction in animal-based products (particularly meat and dairy) may increase the risk of inadequate intake of iron, calcium, vitamin B12, iodine, and zinc in certain populations. A planetary diet can improve nutritional quality; however, its implementation requires careful meal planning to ensure that the reduction in animal-based products does not lead to inadequate micronutrient and macronutrient intake. Particular attention should be given to plant-based foods rich in calcium, iron, zinc, and other essential nutrients, as well as to population-level food fortification measures, such as salt iodization. However, based on the results of the aforementioned studies, the PHD can be nutritionally adequate if the bioavailability of minerals is taken into account, in particular by enhancing it through methods such as soaking, cooking, sprouting, fermenting, and milling plant foods, and by limiting the inhibitory effects of antinutrients.

Notably, plant-based milk alternatives may also support adherence to the PHD, which emphasizes limiting animal-derived products, while also contributing to a reduced environmental impact. Fortified plant-based drinks can provide nutrients such as calcium and vitamins B2 and B12, helping to ensure an adequate dietary intake of these nutrients. Replacing cow’s milk with plant-based alternatives can substantially reduce resource use and greenhouse gas emissions without substantially compromising the overall nutritional value of the diet. However, it is worth noting that classifying fortified plant-based drinks as ultra-processed foods can be misleading and may suggest an adverse effect on health as it fails to take into account their nutritional value and environmental benefits. Moreover, given the potential cost-related barriers to the consumption of fortified plant-based products, improving their affordability and accessibility could support their wider adoption across different population groups.

The planetary diet can simultaneously promote health and environmental protection; however, according to some previous studies, its environmental impact is ambiguous. Studies indicate that greater adherence to the PHD can reduce greenhouse gas emissions—mainly by limiting red meat consumption—while simultaneously increasing the water footprint and land use due to higher intake of foods such as fruits, vegetables, dairy products, and legumes.

Implementing changes is a gradual process that requires time and may be accompanied by various concerns, uncertainties, and questions. In this context, increasing public awareness is essential to support the adoption and acceptance of these changes. Educational institutions can have a significant role in the practical implementation of the planetary diet by combining educational activities with changes in the students’ nutritional environment. Of particular importance is the adoption of a systems approach that accounts for both the environmental impacts of individual interventions and the potential for unintended consequences arising from their implementation. However, from a practical perspective, successful PHD implementation requires more than nutritional recommendations alone. Educational interventions should be combined with improved access to affordable, nutrient-dense foods and gradual dietary changes that preserve familiar meals while increasing plant food intake. In children and adolescents, strategies should account for taste preferences, parental choices, and school meals. Moreover, intervention effectiveness should be assessed in terms of dietary adherence, nutrient intake, affordability, food waste, and long-term sustainability.

There are several limitations to the herein review that should be acknowledged. First, it is important to emphasize the limitations of the available data, such as a small number of participants in the study groups, in some cases a lack of data on food intake in specific populations (e.g., Indonesia), or the use of dietary modeling to align with the PHD, rather than comparing the real and actual habitual diets to the PHD. Moreover, data about food intake were collected using various methods (24 h dietary recall, food frequency questionnaire—FFQ) and the PHDI was calculated using different methods (which makes it difficult to compare its value between different studies), as well as the classification of products into different groups (e.g., peanuts categorized as either nuts or legumes). Furthermore, a narrative review has some limitations itself. There is a possibility for publication selection bias and subjective interpretation of the available data. Moreover, as the search strategy was not fully formalized, the possibility that some relevant studies may have been missed cannot be excluded. Furthermore, the methodological heterogeneity of the analyzed studies and the lack of a quantitative synthesis of the results limit the ability to directly compare their findings and draw definitive conclusions. Taken together, these limitations suggest that the conclusions of the present review should be interpreted as an overview of the current evidence rather than as definitive confirmation of the nutritional and environmental effects of implementing the PHD across all populations. In particular, the heterogeneity of dietary assessment methods, adherence indices, population characteristics, and modeling assumptions limit the extent to which the reported findings can be generalized. The identification of research gaps should therefore guide the development of standardized assessment methods and population-specific interventions, rather than simply encourage further studies of adherence to the PHD.

## 13. Conclusions

The review herein revealed challenges in implementing the Planetary Health Diet, varying across different regions of the globe. Problems in many countries were related to adapting the PHD composition to the customary diet or even the reference diet. The range of required dietary changes depended on gross per capita income. In low-income countries, it seems important to include fortified foods, which could help to ensure an adequate dietary intake of nutrients in these countries. The potential for implementing a balanced diet also lies in promoting the purchase of preferred foods from institutional catering facilities and educating students with various educational profiles. Based on the studies’ results, it is worth noting that adopting a planetary diet would be more effective if initiated during childhood and carried out without drastic changes to dietary composition. The introduction of solid complementary foods during the second half of the first year of life could lay the foundation for future dietary choices and taste preferences. The implementation of changes is a gradual process that requires time and may be accompanied by various concerns, uncertainties, and emerging challenges. Further research is therefore warranted to address these challenges and identify practical solutions that could support the effective implementation of the proposed changes.

## Figures and Tables

**Table 1:** Summary of the studies on the Planetary Health Diet among children and adolescents.

Reference Number	Study Design	Main Findings Associated with the Adherence to the PHD	Limitations
[32]	Germany, children, 4–6 y.o.; model-based dietary assessment; comparative assessment of the original (based on the German Optimized Mixed Diet dietary guidelines) and modified 7-day menu.	PHDI score: 68.24/max. 150 points. The most consistent with PHD (9.9 to 10 out of 10 points): fruits, total vegetables, fish and seafood, vegetable oils, and chicken (and substitutes). The least consistent (zero out of 10 points): tubers and potatoes, dairy, red meat, animal fat, and added sugars. The authors improved the sustainability of the German Optimized Mixed Diet for children and adolescents resulting in an increase in the PHDI score to 81.51 points, without compromising nutrient density.	The study was based on a theoretical 7-day menu not on an actual dietary intake; therefore, findings may not reflect real-world dietary habits of children and adolescents. Socio-economic concerns regarding the affordability of dietary changes.
[33]	Finland, children, 3–6 y.o.; cross-sectional study based on dietary records of Finnish preschool children.	Within the target range: fruits (~66%), chicken and other poultry (~94%), eggs (~67.5%), fish and seafood (100%), legumes (100%), saturated fats (~94.5%), and unsaturated oils (~64.5%). Below the target range: vegetables (~65.5%) and nuts (100%). Above the target range: tubers and starchy vegetables (~79.5%), dairy foods (97%), red meat (100%), beef and lamb (~96.5%), pork (~98%), added sugars (~92.5%)	Cross-sectional design, low participation rate and potential selection bias; participants came from only two regions of Finland; potential inaccuracies in food composition estimates.
[34]	Poland, children, 4–6 y.o.; quantitative observational study, five randomly selected public kindergartens in Wroclaw (Poland); study material: 100 menus covering winter and spring seasons.	The most consistent with the PHD: the average content of vegetables, fruits, dairy products and chicken and other poultry. Below the recommendations: whole grains, fish, legumes, nuts. Above the recommendations: starchy vegetables (potatoes), beef, lamb, and pork, eggs.	Observational design; the study analyzed menus not the actual food intake by children; the findings may not represent all Polish kindergartens; limited seasonal coverage.
[35]	Mexico, adolescents, 12–15 y.o.; a quantitative study with a cross-sectional-descriptive design.	The most consistent with the PHD: the average content of vegetables, low-fat animal protein, saturated oils, whole milk or equivalents, added sugar. Below the recommendations: legumes, unsaturated oils, whole grains. Above the recommendations: high and moderate fat animal protein, tubers or starchy vegetables.	Cross-sectional design; potential bias from self-reported data; the sample was not nationally representative of adolescents in Mexico. *
[36]	Iran, students, 13–18 y.o.; cross-sectional study, multistage stratified cluster random sampling among students from schools in Mashhad (Iran).	PHDI score: 50.79/max. 150 points (scores ranging from 19.66 to 94.46). Lower scores likely were associated with higher consumption of red meat and processed foods, compounded by inadequate intake of nuts and whole grains.	Cross-sectional design; generalizability to healthy Iranian adolescents from Mashhad only; potential influence of unmeasured or inadequately controlled residual confounding; potential recall bias and measurement error due to the FFQ.
[37]	China, adolescents, 11–18 y.o.; an observational study.	The plant-based PHDI (PHDI-green) adherence was defined as consuming at least one daily serving of both vegetables and fruits. The adherence to the PHDI-green was found in 49.5% of the study group.	Observational design does not allow for establishing causal relationships. Self-reported data. Selection bias cannot be fully excluded. Only PHDI-green was assessed.
[38]	Six Latin American countries, adolescents, 10–19 y.o.; secondary cross-sectional analysis.	PHDI score: 42.4/max. 150 points (from 30.3 points in Argentina to 45.1 and 45.3 points in Brazil and Chile, respectively). The highest adherence to the PHD (≥50%): chicken and substitutes, animal fats. The lowest adherence (<20%): nuts and peanuts, whole grains, eggs, fish and seafood, tubers and potatoes, DGV/total vegetables, added sugar.	Cross-sectional design; outdated dietary data (2009–2019, all were collected before the COVID-19 pandemic); methodological differences in urban/rural and socio-economic classifications across countries; some potential confounders were unavailable in the secondary datasets; lack of adjustment for survey stratification and clustering.

PHD—Planetary Health Diet; PHDI—Planetary Health Diet Index; y.o.—years old; FFQ—food frequency questionnaire; DGV—dark green vegetables; *—study limitations were identified by the authors of this review based on an independent assessment, as they were not explicitly reported by the original authors.

**Table 2:** Summary of the studies on the Planetary Health Diet among adults.

Reference Number	Study Design	Main Findings Associated with the Adherence to the PHD	Limitations
[3]	Italy: Modeling study involving the development of an optimized, sustainable dietary pattern based on the PHD. MED_EAT-IT was compared primarily with the previous EAT-IT dietary model.	The MED_EAT-IT diet improved nutritional adequacy and better aligned with the Mediterranean diet. Adopting MED_EAT-IT could reduce the carbon footprint by 31.3% compared with the typical Italian diet. Carbon footprint increased by 12.2%, while water footprint decreased by 6.3% compared with the previous EAT-IT diet.	Theoretical modeling may not reflect real-life dietary behaviors; environmental impact estimates depend on assumptions and production methods; emerging food sources were not included; the model may not meet the needs of all population groups.
[30]	Indonesia: Modeling study comparing 13 dietary scenarios (2 current, 7 optimized, 3 increasingly plant-based, 1 EAT–Lancet).	The EAT–Lancet diet was relatively expensive and had high GHG emissions. Some animal-source foods were necessary to meet nutritional requirements. No dietary scenario simultaneously met all nutritional, health, affordability and environmental goals.	Food availability data may not reflect actual dietary intake; food waste and food losses were estimated using assumptions; the optimization tool did not include all relevant nutrients; food fortification data were unavailable, potentially underestimating micronutrient intake; modeled diets may lack dietary variety and acceptability; environmental assessment covered only GHG emissions and water use.
[39]	Australia: Cross-sectional comparative study using online supermarket price surveys and secondary socio-economic data.	The PHD was significantly cheaper and more affordable than the typical Australian diet across metropolitan Australia and all socio-economic groups. It accounted for 13% of median disposable household income, compared with 16% for the typical diet.	The study included only metropolitan areas in Australia; food prices were collected from only one supermarket chain; outdated data on dietary habits; limited household representation; no consideration of special dietary requirements; the cost of eating out was excluded from the analysis; consumer acceptability and practical barriers to adopting the PHD were not assessed.
[40]	USA, young adults, 22.1 ± 2.0 y.o.; cross-sectional study.	PHD score: 4.1 ± 1.4/max. 14 points. Within the target range: vegetables (69.7%), fruits (89.8%), added fats (94.3%); below the target range: whole grains (87.7%), potatoes (59.1%), dairy products (58.6%), chicken and other poultry (58.9%), fish (91.4%), beans, lentils and peas (95.3%), soya (96.3%), nuts (98.6%); above the target range: beef, lamb and pork (71.7%), added sweetener (87.8%).	Cross-sectional design; participants were recruited from one metropolitan area in Minnesota; the FFQ may not accurately capture dietary intake (limited assessment of plant-based proteins, such as soya).
[41]	Argentina: Quantitative, model-based analysis; the sources of data: existing national dietary survey data, official food prices and household income data; model diets developed for a reference household (composed of four people: a 45 y.o. man and woman, a 7 y.o. girl, and a 14 y.o. boy).	The most consistent with the PHD: dairy foods; below the recommendations: fruits, vegetables, tubers or starchy vegetables, grains, legumes, unsaturated oils—nuts and seeds; above the recommendations: animal protein sources, discretionary foods.	Theoretical dietary models, the study did not reflect the actual consumption of the population; the study used food price data from Greater Buenos Aires, which may not reflect prices in other regions of Argentina.
[42]	Longitudinal comparative cost analysis. The study aimed to assess the feasibility and cost of implementing an EAT–Lancet-based Healthy and Sustainable Food Basket in Chile compared with the standard Basic Food Basket used to define the poverty line.	The Healthy and Sustainable Basic Food Basket, based on EAT–Lancet recommendations, was on average 13.9% more expensive than the standard Basic Food Basket in Chile between 2014 and 2023. However, it provided approx. 2001 kcal/day, with a balanced distribution of macronutrients.	The EAT–Lancet-based basket was compared with Chile’s standard Basic Food Basket, designed to provide approximately 2000 kcal/day, rather than with the average national diet. Limited comparability with other studies.
[43]	Iran (Teheran), adults ≥60 y.o.; cross-sectional study.	PHDI score: women 57.81 ± 9.25 and men 55.46 ± 9.71/max. 150 points. Higher adherence to the PHDI was associated with reduced dietary costs.	Cross-sectional design; potential bias associated with dietary intake assessment using an FFQ (recall bias, a limited list of food items); food consumed outside the home was not included (this may have led to inaccurate dietary cost estimates); simplified food cost estimates; the study did not consider food waste; potential residual confounding.
[44]	A comparative, cross-country analysis of dietary patterns and environmental impacts across 37 African countries.	The average score across Africa was 38.1/max. 150 points; North Africa vs. Southern Africa (51.9 vs. 28.9 points). Low adherence to the EAT–Lancet diet with regional differences; greater adherence to the EAT–Lancet diet could reduce greenhouse gas emissions; however, it could also increase blue water consumption and cropland use in some regions.	The analysis was based on aggregated country-level data; only selected environmental indicators were assessed, the modeling approach does not establish causal relationships; the study design does not allow for establishing causal relationships. *
[45]	Sweden: Quantitative modeling study, reference diet from the scenario narratives and current dietary data in Sweden.	The most consistent with the PHD: vegetable oils; below the recommendations: cereals, fruits, pulses, treenuts, vegetables; above the recommendations: blue food, eggs, meat, starchy roots, sweeteners.	The study used modeled dietary scenarios, not the actual dietary intake; uncertainty in biodiversity assessment; limited assessment of local biodiversity; uncertainty regarding emerging food technologies.

PHD—Planetary Health Diet; PHDI—Planetary Health Diet Index; y.o.—years old.; GHG—greenhouse gas; *—study limitations were identified based on the available information, as the full text of the publication was unavailable, the first two limitations are based on the study methodology, while the third can be inferred from the study design.

## Data Availability

No new data were created or analyzed in this study. Data sharing is not applicable to this article.

## Supplementary Materials

The following supporting information can be downloaded at https://www.mdpi.com/article/10.3390/foods15213813/s1. Figure S1: Flowchart with the strategy of literature search.

## Abbreviations

The following abbreviations are used in this manuscript:

FAO	Food and Agriculture Organization of the United Nations
GBD	Global Burden of Disease
GDP	Gross Domestic Product
GHG	Greenhouse Gas
HAQ	Healthcare Access and Quality
NHANES	National Health and Nutrition Examination Survey
PHD	Planetary Health Diet
PHDI	Planetary Health Diet Index
USA	United States of America
